# Cooperation between myofibril growth and costamere maturation in human cardiomyocytes

**DOI:** 10.3389/fbioe.2022.1049523

**Published:** 2022-11-01

**Authors:** Huaiyu Shi, Chenyan Wang, Bruce Z. Gao, James H. Henderson, Zhen Ma

**Affiliations:** ^1^ Department of Biomedical & Chemical Engineering, Syracuse University, Syracuse, NY, United States; ^2^ BioInspired Institute for Materials and Living Systems, Syracuse University, Syracuse, NY, United States; ^3^ Department of Bioengineering, Clemson University, Clemson, SC, United States

**Keywords:** costameres, sarcomeres, focal adhesions, cardiomyocytes, human induced pluripotent stem cells

## Abstract

Costameres, as striated muscle-specific cell adhesions, anchor both M-lines and Z-lines of the sarcomeres to the extracellular matrix. Previous studies have demonstrated that costameres intimately participate in the initial assembly of myofibrils. However, how costamere maturation cooperates with myofibril growth is still underexplored. In this work, we analyzed zyxin (costameres), α-actinin (Z-lines) and myomesin (M-lines) to track the behaviors of costameres and myofibrils within the cardiomyocytes derived from human induced pluripotent stem cells (hiPSC-CMs). We quantified the assembly and maturation of costameres associated with the process of myofibril growth within the hiPSC-CMs in a time-dependent manner. We found that asynchrony existed not only between the maturation of myofibrils and costameres, but also between the formation of Z-costameres and M-costameres that associated with different structural components of the sarcomeres. This study helps us gain more understanding of how costameres assemble and incorporate into the cardiomyocyte sarcomeres, which sheds a light on cardiomyocyte mechanobiology.

## 1 Introduction

Functioning as the mediators of cell-extracellular matrix (ECM) interactions, focal adhesions are responsible for sensing and transducing extracellular signals to regulate cell behaviors and functions (González-García et al., 2010; Seo et al., 2013; Yip et al., 2018; Natale et al., 2019; Fedele et al., 2020). Cardiomyocytes exhibit two distinct focal adhesion structures: periphery focal adhesions (pFAs) close to the cell perimeter, which are laterally parallel along the myofibrils; and costameres registered with sarcomeres, which are transverse across the myofibrils. Costameres, as striated muscle-specific cell adhesion structures, were first described as vinculin-containing structures located between the sarcolemma and myofibrils (Pardo et al., 1983). Except for vinculin, costameres have been proved to share similar components with pFAs (e.g., integrins (McDonald et al., 1995), FAK (Kovačič-Milivojević et al., 2001), and talin (Belkin et al., 1986)). This multiprotein architecture serves as the linkages between sarcomeres and ECMs, thus regulates the contractile functions of muscular tissues (Lyon et al., 2015). Previous studies have shown that muscle atrophy resulting from the reduction in mechano-loading might be accompanied by decreased expression of costamere components (Li et al., 2013; Ruoss et al., 2018; Gorza et al., 2021). In addition, costamere formation is also affected by the mechanical properties of ECM (Galie et al., 2013). These studies indicated that costameres are highly regulated by the extracellular mechanical microenvironment, and responsible for transducing contractile force from myofibrils to ECMs. Despite extensive studies focusing on focal adhesions in general, behaviors of costameres are less investigated, leaving unanswered questions of how costameres interact with myofibrils to mediate cardiomyocyte contraction.

Myofibrils make up the contractile machinery of individual cardiomyocytes consisting of serially arranged sarcomeres comprising thick filaments (myosin and associated proteins), thin filaments (actin and associated proteins), and Z-discs (α-actinin). Myofibrillogenesis was proposed to occur *via* a three-step process: pre-myofibrils to nascent myofibrils to mature myofibrils (Sanger et al., 2006; Sanger et al., 2010; Sanger et al., 2017). Pre-myofibrils are composed of mini-sarcomeres that are bordered by Z-bodies containing muscle α-actinin. The bipolar arrangement of the actin filaments in the mini-sarcomeres are held together by bipolar non-muscle myosin II filaments. Nascent myofibrils are formed by adding titin molecules and overlapping thick muscle myosin filaments to the former pre-myofibrils. The mature myofibrils result from the elimination of non-muscle myosin II, the alignment and fusion of several Z-bodies to form Z-bands, and the alignment of muscle myosin II filaments to form A-bands. Furthermore, late assembling proteins are added to form the mature myofibrils: telethonin, a titin binding protein in the Z-bands; muscle myosin II binding C protein in the cross-bridge regions of the A-bands; the incorporation of myomesin into the middle of the A-bands to form M-bands.

**FIGURE 1:**
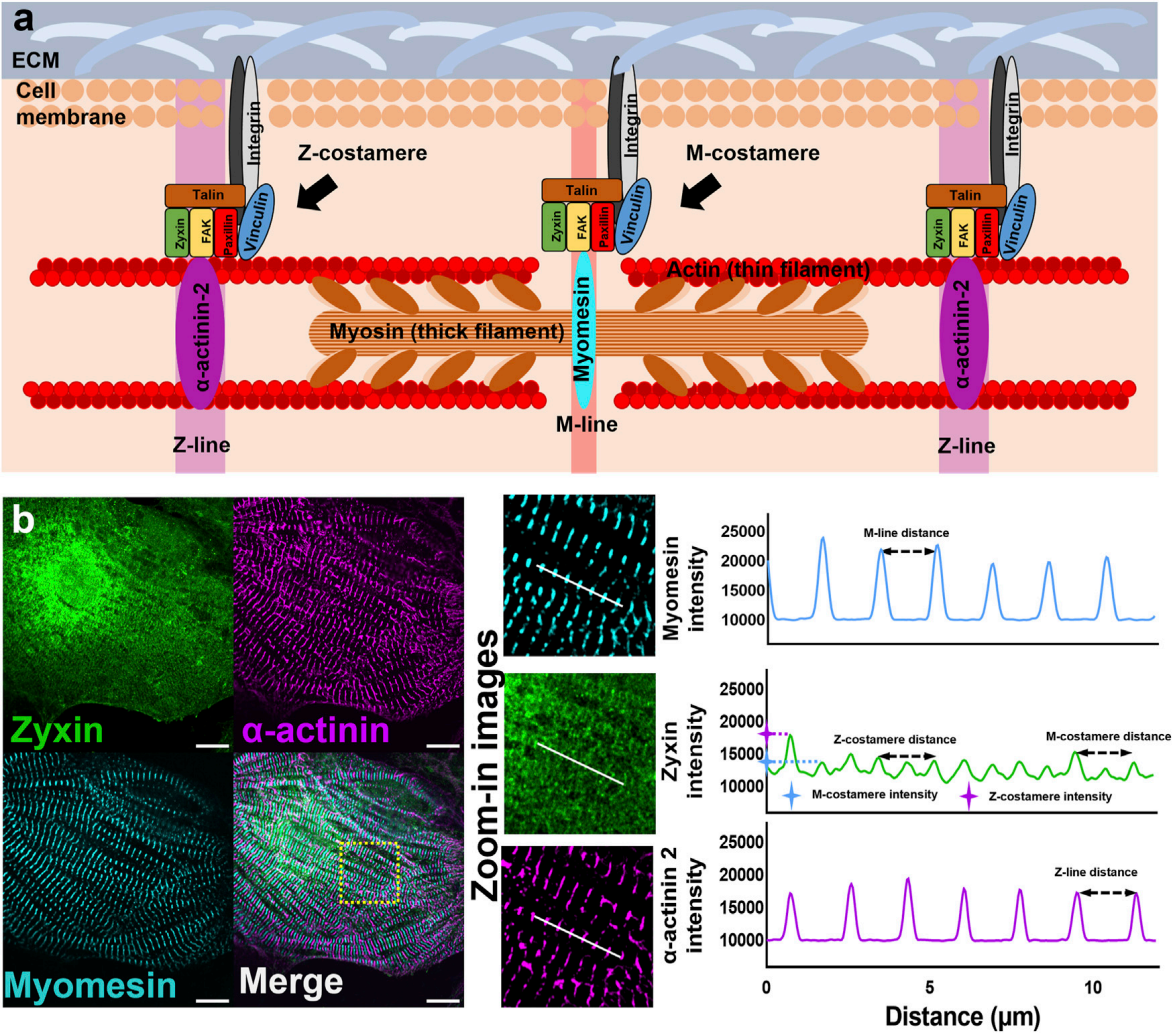
Myofibrils and costameres in cardiomyocytes **(A)** Schematics showing the components of sarcomeres and costameres in cardiomyocytes. **(B)** Fluorescent images of Z-lines (α-actinin), M-lines (myomesin), and costameres (zyxin). Intensity profiles to demonstrate the measurement of Z/M-line distance, Z/M-costamere distance, and Z/M-costamere intensity. Scale bar: 10 μm.

During this process, the costameres provide anchors for the initial recruitment and stability of the sarcomeres (Myhre and Pilgrim, 2012). Particularly, costameres are registered with the Z-discs (Z-costameres) and the central M-line of the A-band (M-costameres), showing a striated rib-like pattern upon becoming mature costameres (also called costamere maturation) (Figure 1A) (di Mauro et al., 2009). However, the process of costamerogenesis and particularly how costameres assemble and mature into the striated pattern is poorly studied. Early work found complete devastation of mature costamere structures associated with a decreased proportion of mature myofibrils in FAK-inhibited cardiomyocytes, which led to a hypothesized model discussing the interdependence between costamerogenesis and myofibrillogenesis (Quach and Rando, 2006). This theory was reinforced by a recent investigation in which the cardiomyocyte contractile force was impaired in a vinculin knockout cell line and myofibril-flawed cell lines. Neither mature costameres nor organized myofibrils were found in these contraction-deficient cells (Chopra et al., 2018). These studies elucidated that the formation of costameres was driven by the contractile force, which in return played a critical role in maintaining the contractions of cardiomyocytes. Most of these early studies on costamere biology focused on the Z-costameres, while biological role and behaviors of M-costameres are much less investigated (Porter et al., 1992; Hresko et al., 1994; Anastasi et al., 2003). More importantly, how M-costameres and Z-costameres assemble and incorporate in the sarcomeres during costamerogenesis remains unclear.

In this study, we utilized human-induced pluripotent stem cell-derived cardiomyocytes (hiPSC-CMs) to study the assembly and maturation of costameres during cell attachment and spreading. Compared to adult cardiomyocytes containing mature myofibrils and costameres in both peripheral area and center area of the cells, hiPSC-CMs provide a developmental framework to investigate the process from *de-novo* assembly to maturation of costameres and myofibrils *in vitro*. Herein, we focus on the maturation of myofibril-costamere cytoarchitecture into striated patterns, instead of the maturation of stem cell-derived embryonic-like cardiomyocytes into adult-like phenotypes. To understand how costameres were assembled and incorporated with the sarcomeres, we characterized the behavior of costameres and myofibrils during the initial stage of cell attachment from Hour four to Hour eight post-hiPSC-CMs seeding. To examine the maturation process of costameres and myofibrils, we characterized the behavior of costamere and myofibril during the later stage of cell spreading at Hour 8, Hour 12, Hour 24, and Hour 48 post-hiPSC-CMs seeding.

Zyxin was used to track the distribution of costameres within hiPSC-CMs. Z-costameres and M-costameres were distinguished by the colocalization with sarcomere α-actinin (Z-line protein) and myomesin (M-line protein) respectively. By temporal sampling the hiPSC-CMs after cell seeding, we quantified the activities of Z-costameres and M-costameres from *de-novo* assembly to the maturation during costamerogenesis. We also tracked the behavior of M-lines and Z-lines to profile the coordination between myofibrillogenesis and costamerogenesis. We found that the *de-novo* assembly of M-costamere and Z-costamere proceeded simultaneously while the maturation of Z-costameres and M-costameres were asynchronous. Moreover, we grew hiPSC-CMs on stiffness-tunable polyacrylamide hydrogel to investigate how costamere formation could be affected by the substrate mechanical properties. This study provides detailed evidence to fill in the knowledge gap of how costameres interact with myofibrils to maintain the contractile functions of cardiomyocytes, which should enable future progress in understanding the process of cardiomyocyte mechanobiology *in vitro*.

## 2 Method and materials

### 2.1 hiPSC-CMs differentiation and purification

hiPSC-CMs were differentiated from mEGFP-tagged ACTN2 WTC hiPSC line edited with CRISPR/Cas9 technology (Coriell Institute, Ca# AICS-0075-085), which have fluorescent reporter on sarcomere α-actinin under 488 nm excitation in the differentiated hiPSC-CMs. The hiPSCs were cultured in Essential 8 (E8) media (Thermo Fisher Scientific, Ca# A1517001) with media refreshment every 24 h on 6-well plates pre-coated with Geltrex (Thermo Fisher Scientific, Ca# A1413302). The cell passaging was performed every 3 days at the seeding density of 2.5 × 104 cells/cm^2^ in the E8 media supplemented with ROCK inhibitor (10 μM, Y-27632; BioVision, Ca# 1994) for the first 24 h. The differentiation procedures have been detailed in our previous publications (Hoang et al., 2018). Briefly, differentiation was initialized by modulating the WNT pathway using two small molecules, GSK3 inhibitor (6 μM, CHIR99021; Stemgent, Ca# 04-0004) and WNT inhibitor (5 μM, IWP4; Stemgent, Ca# 04-0036) in RPMI 1640 media (Thermo Fisher Scientific, Ca# A1517001) with B27 supplement minus insulin (Thermo Fisher Scientific, Ca# A1895601) (RPMI-B27-I). Then, cells were cultured in RPMI 1640 media with B27 complete supplement (Thermo Fisher Scientific, Ca# 17504044) (RPMI-B27 + C) until Day 16 for purification procedures. The differentiated hiPSC-CMs were disassociated and replated on Geltrex pre-coated 6-well plates with RPMI-B27 + C media supplemented with ROCK inhibitor. After 2-day recovery in RPMI-B27 + C media, cells were purified in a media of DMEM without glucose (Thermo Fisher Scientific, Ca# 11966-025) supplemented with NEAA (Thermo Fisher Scientific, Ca# 11140050), GlutaMAX (Thermo Fisher Scientific, Ca# 35050061) and 4 mM lactate (Sigma Aldrich, Ca# L7022) for 6 days with media refreshment every 2 days. The purified hiPSC-CMs were maintained in RPMI-B27 + C media refreshed every 2 days.

### 2.2 Cell seeding and sampling

Differentiated hiPSC-CMs were dissociated from the 6-well plates and seeded on the substrates pre-coated with Geltrex. To investigate the *de-novo* formation of costameres and myofibrils at the initial stage of hiPSC-CMs attachment, we sampled the hiPSC-CMs hourly during Hours 4-8 post cell seeding. To investigate the activities of costameres and myofibrils at the later stage of hiPSC-CMs spreading, we sampled the hiPSC-CMs at Hours 8, 12, 24, and 48 post cell seeding. We then performed immunofluorescence staining on the selected samples for further measurements. To study how costameres and myofibrils would be affected by extracellular mechanical properties, we seeded hiPSC-CMs on the substrates pre-coated by PA hydrogels of varying stiffness (see *Traction force microscopy analysis*). At 48 h, the hiPSC-CMs were transferred to an on-stage incubator and motion videos for traction force microscopy were recorded using a Nikon Eclipse Ti microscope with Zyla 4.2 PLUS sCMOS camera. After the video recording, the hiPSC-CMs were fixed and stained for further measurements on costameres and sarcomeres.

### 2.3 Immunocytochemistry

Cell samples were fixed in 4% PFA solution for 20 min, permeabilized with 0.2% triton solution for 5 min, and blocked with 2% bovine serum albumin (BSA; Sigma Aldrich, Ca# A8022) for 30 min. The fixed samples were then incubated in primary antibody solution (Supplementary Table S1) for 2 h, washed with PBS three times, and incubated with secondary antibodies for 2 h. After three times of PBS washing, the cell samples were ready to image. The bright-field and epifluorescence microscopy was performed on a Nikon Eclipse Ti microscope with Zyla 4.2 PLUS sCMOS camera. The confocal microscopy was performed on a Zeiss LSM 980 with Airyscan two confocal microscope.

### 2.4 Quantification of costamere and myofibril properties

To analyze the myofibril structure, the M-line component myomesin and Z-line component α-actinin were stained and imaged using the confocal microscope. For the myofibril characterizations, the distance between two adjacent Z-lines (Z-line distance) and Z-line density were quantified to examine the Z-disc assembly. The distance between two adjacent M-lines and M-line density were quantified to examine the M-band assembly. The Z-line length and M-line length were measured to examine the maturation of myofibrils. M-line distance, Z-line distance, Z-line length, M-line length, Z-line number and M-line number were characterized using a Matlab-based software (Morris et al., 2020). Cell area was measured using Fiji ImageJ. Z-line density and M-line density were calculated as follows:

$$Z-line\;density=\frac{M-line\;number\;\left(count\right)}{Cell\;area\;\left({\mu m}^{2}\right)}$$

$$M-line\;density=\frac{M-line\;number\;\left(count\right)}{Cell\;area\;\left({\mu m}^{2}\right)}$$

The analysis of costameres was performed using the fluorescent images of costamere component zyxin. Fiji ImageJ was used to plot the intensity profile of costameres and myofibrils. Based on the intensity profiles, Z-costameres were identified by the colocalization of costamere peaks with actinin peaks, while the M-costameres were identified by the co-localization of costamere peaks with myomesin peaks (Figure 1B). To characterize the assembly of costameres, the distances between two adjacent Z/M-costameres were characterized by the distance between two fluorescent peaks from the intensity profiles of Z/M costameres. To characterize the maturation of costameres, the intensity of costameres was characterized by the amplitude of the fluorescent peak and then normalized by the intensity value of fluorescent background from the regions without cells or debris.

### 2.5 Traction force microscopy analysis

To analyze how costameres respond to different substrates stiffnesses, the hiPSC-CMs were cultured on polyacrylamide (PA) hydrogels with stiffness of 10 kPa and 40 kPa (Tse and Engler, 2010; Simmons et al., 2013). The fabrication of PA hydrogel followed a standard protocol (Ribeiro et al., 2016), starting at the preparation of prepolymer solution, made of acrylamide (BIO-RAD Ca# 1610140), bis-acrylamide (BIO-RAD Ca# 1610142), 35 mM HEPES buffer (SIGMA Ca# 7365-45-9), 0.1% w/v ammonium persulfate (BIO-RAD Ca# 1610700), 0.1% v/v N′-Tetramethylethylenediamine (TEMED, BIO-RAD Ca# 161-0801) and Milli-Q water. The stiffness of the hydrogels is mediated by altering the concentration of acrylamide (40 kPa, 8%; 10 kPa, 10%) and bisacrylamide (40 kPa, 0.48%; 10 kPa, 0.1%) used in the prepolymer solution (Ribeiro et al., 2015). To enable the measurement of hiPSC-CM force generation, fluorescent microbeads (Thermo Fisher Scientific, Ca# F8805) were incorporated to the prepolymer solution with a final concentration of 6×10^9^ microbeads/mL. To create a PA hydrogel with flat surface, 15 ml of prepolymer solution was dripped on a glass slide precoated with fibronectin. A glass coverslip, silane-treated with 0.4% 3-(trimethoxysilyl) propyl methacrylate (SIGMA Ca# 2530-85-0) for 1 h, was gently placed on the top of the prepolymer solution drop. After 15 min polymerization, the hydrogel together with the glass slide was immersed in phosphate-buffered saline (PBS) solution for 1 h to allow the hydration of hydrogel. After the hydration, the PA hydrogel-coated cover glass was gently removed from the glass slide using a razor blade. Before cell seeding, the PA hydrogel was immersed in PBS solution overnight to further rinse out unreacted monomer.

For traction force microscopy, videos containing dispersed fluorescent beads were captured under 330 nm excitation with a frame rate of 18 fps (Supplementary Movie S1). The videos were analyzed using a Matlab-based software to generate the plots of hiPSC-CMs contractile force (Supplementary Movie S2) and contractile energy (SupplementaryMovie S3) (Sergé et al., 2008; Mandal et al., 2014; Kumari et al., 2019; Kumari et al., 2020). These plots were applied to an in-house MATLAB script to calculate the maximum contractile energy, maximum upstroke power, maximum upstroke force (Ma et al., 2018).

### 2.6 Sarcomere shortening analysis

To investigate how different substrate stiffness could affect the contraction of hiPSC-CMs, the sarcomere shortening was measured by tracking the position of individual Z-lines in a beating video. The videos containing the Z-line movements in live hiPSC-CMs were captured under the excitation of 488 nm excitation with a frame rate of 20 fps (Supplementary Movie S4) and analyzed using a Python 3.6-based algorithm to identify the sarcomere shortening percentile, sarcomere contraction duration, and sarcomere relaxation duration.

### 2.7 Statistical analysis

One-way ANOVA with Tukey multiple t-test was used to compare the difference among groups. The statistical significance was determined as *p*-value <0.05 (*), <0.01(**), <0.001 (***) and <0.0001 (****), respectively. For each condition, more than 20 hiPSC-CMs were selected for quantification. In myofibril measurement, all clean Z-line and M-line within individual hiPSC-CMs were measured for each condition. In costamere analysis, four different locations containing myofibrils were randomly selected in each hiPSC-CM for measurement. Data visualization, including box plots showing the minimum, maximum, median, and 25th and 75th percentiles, was performed by software GraphPad Prism 6.

## 3 Results

### 3.1 Assembly of costameres in hiPSC-CMs during cell attachment

**FIGURE 2:**
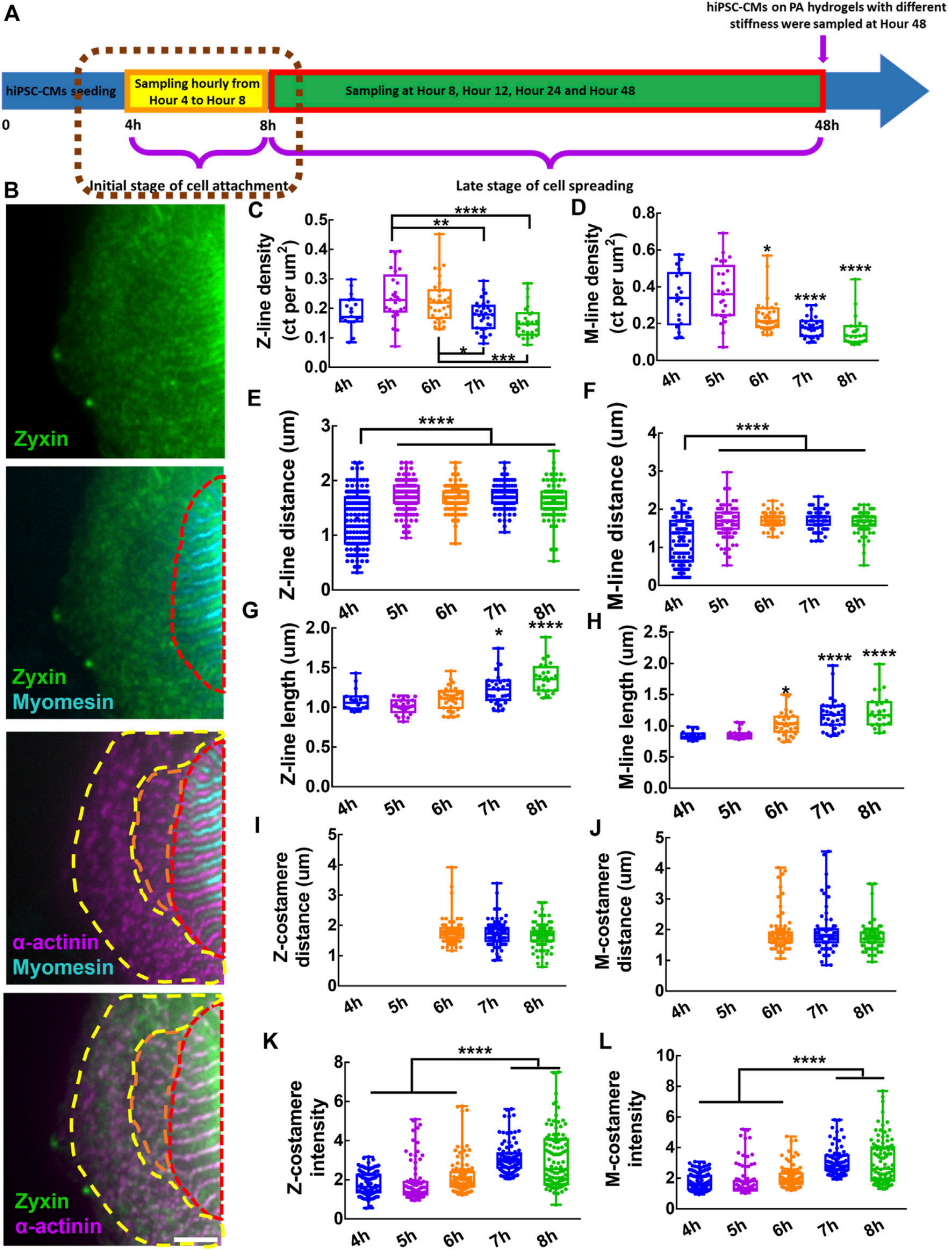
Assembly of costameres in hiPSC-CMs during cell attachment. **(A)** Schematics showing the sampling of hiPSC-CMs in the initial stage of cell attachment. **(B)** Fluorescent images of Z-lines, M-lines and costameres. The region containing mature costameres and mature myofibrils is indicated by a red dashed line. Nascent myofibrils with striated-like Z-lines colocalized with pre-costameres are indicated by an orange dashed line. Pre-myofibrils with Z-bodies colocalized with pre-costameres in the cell peripheral region are indicated by a yellow dashed line. Scale bar: 5 μm. To characterize the assembly and growth of myofibrils within hiPSC-CMs during cell attachment, we measured the **(C)** Z-line density, **(D)** M-line density, **(E)** Z-line distance, **(F)** M-line distance, **(G)** Z-line length and **(H)** M-line length. To track the assembly of costameres, we quantified the **(I)** Z-costamere distance **(J)** M-costamere distance **(K)** Z-costamere intensity and **(L)** M-costamere intensity. *p < 0.05*,* **p < 0.01, ***p < 0.001 and ****p < 0.0001.

We investigated the formation of myofibrils and costameres at the initial stage of cell attachment by sampling hiPSC-CMs hourly from Hour four to Hour eight post cell seeding (Figure 2A). From the confocal fluorescent images of zyxin-actinin-myomesin co-staining, we found that the zyxin pre-costameres colocalized with both pre-myofibrils containing Z-bodies in the peripheral region of hiPSC-CMs and the nascent myofibrils containing striated-like Z-lines but no M-lines. The striated mature costameres only existed in the mature myofibrils containing striated Z-lines and M-lines (Figure 2B). During hiPSC-CMs attachment, Z-bodies aggregated in the peripheral region and close to the mature myofibrils with striated Z- and M-lines at Hour four and Hour 5, when the pre-costameres showed no obvious striated repeating pattern (Supplementary Figure S1). From Hour six to Hour 8, the striated sarcomeric Z-lines and M-lines were colocalized in both the peripheral and center areas of the cells, indicating the maturation of myofibrils. Moreover, during Hours 6–8, the Z-costameres and the M-costameres were assembled to a detectable level, which showed the striated pattern colocalizing with the mature myofibrils (Supplementary Figure S1).

To study the myofibril assembly and growth, we characterized the properties of Z-lines and M-lines of the hiPSC-CMs, including distance, length, and density from fluorescent images of α-actinin and myomesin. Both Z-line density and M-line density had a trend of an increase from Hour 4 to Hour 5, followed by a clear decrease from Hour 5 to Hour 8, with a peak value at Hour 5 (Figures 2C,D). These results indicated a fast *de-novo* assembly of Z-lines and M-lines at Hour 5, which was slowed down during Hours 6–8 due to a rapid increase of cell area (Supplementary Figure S2). We also found that both Z-line distance and M-line distance during Hours 5–8 were significantly longer than at Hour 4, indicating a progressive transition from pre-myofibrils to nascent myofibrils during the initial stage of cell attachment would increase the sarcomere distance (Figures 2E,F). Using Z-line length and M-line length as sarcomere maturity measurement, we found that Z-line length increased significantly during Hours 7-8, while the M-line length increased significantly during Hours 6–8 (Figures 2G,H). These results suggested the growth of Z-lines and M-lines starts immediately after *de-novo* assembly of the sarcomeres.

To examine the assembly of Z-costameres and M-costameres, we measured the Z-costamere distance, M-costamere distance, Z-costamere intensity, and M-costamere intensity based on zyxin staining colocalized with α-actinin and myomesin. From the fluorescent images, we only found clear Z-costameres and M-costameres appearing at Hour 6, which was later than the formation of striated myofibrils starting at Hour 4 (Supplementary Figure S1). No significant difference was found in Z-costamere distance and M-costamere distance among different time points (Figures 2I,J). Though the observable costameres occurred at Hour 6, we found that zyxin accumulated to form pre-costameres at the positions of Z-lines and M-lines and that the pre-costameres’ fluorescent intensity could be quantified as early as the Hour 4-5 time points. The results showed that the fluorescent intensity of both Z-costameres and M-costameres at Hour 7-8 was significantly higher than the ones at Hour 4–6, indicating that fully assembled costameres had more zyxin components than the *de-novo* clusters of early costameres (Figure 2k,L). From Hour 4 to Hour 8, no significant difference on the zyxin fluorescent intensity was found between Z-costameres and M-costameres (Supplementary Figure S2), suggesting that the zyxin was equally recruited to the Z-costameres and M-costameres during hiPSC-CMs attachment. We also found that there was no significant difference in distance between Z-costameres and Z-lines (Supplementary Figure S2), while the M-line distance was significantly shorter than the M-costamere distance during Hours 6–8 (Supplementary Figure S2). These results suggested that the Z-lines intimately associate with Z-costameres during myofibril growth and maturation, while some M-lines might not be registered to M-costameres in the middle of the myofibrils (Supplementary Figure S2).

### 3.2 Maturation of costameres during cell spreading

**FIGURE 3:**
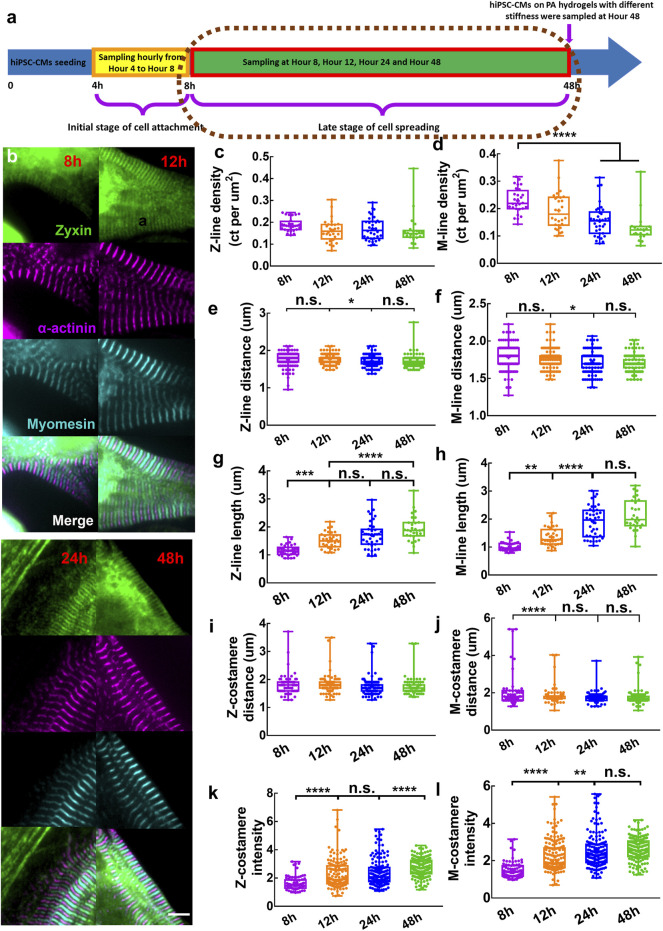
**(A)** Schematics showing sampling of hiPSC-CMs in the late stage of cell spreading. **(B)** Zoom-in images showing mature Z/M-costameres together with mature myofibrils within the hiPSC-CMs. Scale bar: 5 μm. To track the myofibril growth within hiPSC-CMs during cell spreading, we measured the **(C)** Z-line density, **(D)** M-line density, **(E)** Z-line distance, **(F)** M-line distance, **(G)** Z-line length and **(H)** M-line length. To track the costamere growth during cell spreading, we quantified **(I)** Z-costamere distance **(J)** M-costamere distance **(K)** Z-costamere intensity and **(L)** M-costamere intensity. *p <0.05, **p <0.01, ***p <0.001 and ****p <0.0001.

To elucidate how costamerogenesis cooperates with myofibrillogenesis in a later stage of cell spreading, we characterized the temporal behavior of myofibrils and costameres at Hour 8, Hour 12, Hour 24, and Hour 48 post cell seeding (Figure 3A). The myomesin-containing M-lines were in the center of two adjacent actinin-containing Z-lines, showing an alternative striated pattern of the mature myofibrils observed from all the timepoints (Supplementary Figure S3). Z-costameres and M-costameres showed a striated pattern of mature costameres (Figure 3B). We found a progressive increase of cell area, reaching a significant larger area at Hour 48 comparing to Hour 8 (Supplementary Figure S4). By tracking the myofibril growth during cell spreading, we found Z-line density maintained a stable level, indicating a relative fast assembly of Z-lines for myofibril growth to meet the increase of cell area (Figure 3C). In contrast, we found a significant decrease of M-line density from Hour eight to Hour 48 (Figure 3D), suggesting the M-line assembly was slower than the Z-lines, indicating the myofibril maturation was delayed during the cell spreading. The Z-line distance and M-line distance showed a consistent decreasing trend from Hour eight to Hour 24, which also indicated a progressive formation of new Z-lines and M-lines within the myofibrils (Figures 3E,F). Meanwhile, we found that the Z-line length showed a rapid increase from Hour eight to Hour 12, followed by a slow growth from Hour 12 to Hour 48 (Figure 3G). The M-line length showed a similar increasing trend from Hour eight to Hour 48, but the rapid growth occurred from Hour 12 to Hour 24 (Figure 3H). These results indicated that the maturation of the M-lines was later than the Z-lines during myofibrillogenesis.

We quantified the features of Z-costameres and M-costameres from Hour eight to Hour 48 post cell seeding to investigate their distinct roles during costamerogenesis. The Z-costamere distance showed no significant difference from Hour eight to Hour 48, suggesting the assembly of Z-costameres had been completed before Hour 8 (Figure 3I). In contrast, the M-costamere distance at Hour eight was significantly longer than Hour 12–48 (Figure 3J), indicating a delayed assembly of the M-costameres than the Z-costameres. We found that the intensity of both Z-costameres and M-costameres showed an increasing trend Hour eight to Hour 48. Interestingly, the accumulation of zyxin in Z-costameres and M-costameres demonstrated an alternating pattern of increasing fluorescent intensity. A significant increase of M-costamere intensity occurred between Hour 12 and Hour 24, while the Z-costamere intensity was unchanged. While the intensity of M-costameres reached a stable level at Hour 24, a significant increase of Z-costamere intensity was observed during Hours 24–48 (Figures 3K,L). These results revealed distinct costamerogenesis processes between Z-costameres (early formation but late maturation) and M-costameres (late formation but early maturation). We also found a significantly higher intensity of Z-costameres than of M-costameres at Hour 48, indicating the abundance of Z-costameres in long-term culture of hiPSC-CMs (Supplementary Figure S4). For the distance comparison between myofibrils (Z-lines and M-lines) and costameres (Z-costameres and M-costameres), we found similar results as at earlier timepoints (Hour 4–8). Z-line distance and Z-costamere distance showed no significant difference (Supplementary Figure S4). We found a larger M-costamere distance than M-line distance at Hour 8 (Supplementary Figure S4), which was consistent with early timepoints (Supplementary Figure S2), but such difference was no longer obvious in the later timepoints, indicating that M-lines were fully registered to the M-costameres during myofibril maturation.

### 3.3 Costamere maturation on the substrates of different stiffness

**FIGURE 4:**
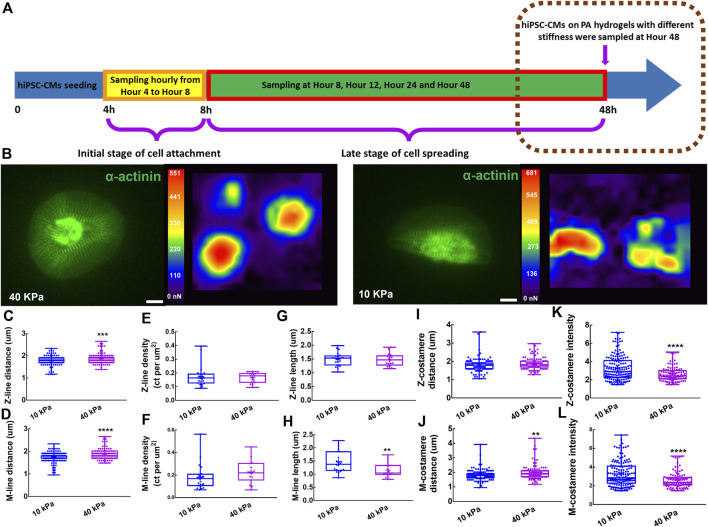
Costameres of hiPSC-CMs on different substrates. **(A)** Schematics showing the experiment of hiPSC-CMs seeding on PA hydrogels with different stiffness. **(B)** hiPSC-CMs cultured on the PA hydrogels with 10 kPa and 40 kPa modulus were analyzed for their mechanical output based on traction force microscopy. Scale bar: 10 μm. To characterize the responsiveness of myofibrils to different substrate stiffness, we measured the **(C)** Z-line density, **(D)** M-line density, **(E)** Z-line distance, **(F)** M-line distance, **(G)** Z-line length, **(H)** M-line length. To investigate the behaviors of Z-costameres and M-costameres of hiPSC-CMs on different substrates, we quantified the **(I)** Z-costamere distance **(J)** M-costamere distance **(K)** Z-costamere intensity and **(L)** M-costamere intensity. *p <0.05, **p <0.01, ***p <0.001 and ****p <0.0001.

To investigate the role of costameres in sustaining the contractile function of hiPSC-CMs, we seeded hiPSC-CMs on PA hydrogel with moduli of 10 kPa and 40 kPa, respectively, for 48 h (Figure 4A). No significant difference was found on cell area of the hiPSC-CMs on the different substrates (Supplementary Figure S5). We performed traction force microscopy (TFM) for the hiPSC-CMs (Figure 4B) and found that the hiPSC-CMs on 40 kPa PA hydrogels showed impaired contractions, manifesting as significant deterioration of maximum contractile energy, maximum upstroke power, and maximum upstroke force (Supplementary Figure S5). When we characterized the responsiveness of myofibrils of hiPSC-CMs to different substrate stiffnesses, we found that the Z-line distance and M-line distance were increased significantly on the 40 kPaPA hydrogel compared to the 10 kPa PA hydrogel (Figures 4C,D). No significant difference was found in Z-line length Z-line density within the hiPSC-CMs between the 10 kPa and 40 kPa groups (Figures 4E,G), suggesting the maturity of the Z-lines was unchanged in response to the substrates with different stiffness. However, M-line length of the hiPSC-CMs on the 40 kPa substrate was significantly shorter than those of cells on the 10 kPa substrate, suggesting the M-lines were more sensitive to extracellular mechanical microenvironment (Figure 4H). In sarcomere shortening analysis, we found the sarcomere shortening was significantly decreased on the 40 kPa PA hydrogel compared to the 10 kPa PA hydrogel. No significant difference was found in sarcomere contraction duration and sarcomere relaxation duration (Supplementary Figure S5). When we investigated the behaviors of Z-costameres and M-costameres of hiPSC-CMs on different substrates, we found that Z-costamere distance showed no significant difference between the 10 kPa group and 40 kPa group (Figure 4I). However, a significantly longer M-costamere distance was observed in the 40 kPa group, which was consistent with the increase in M-line distance (Figure 4J). We found that both Z-costamere intensity and M-costamere intensity were significantly lower in hiPSC-CMs on the 40 kPa hydrogel compared to the ones on the 10 kPa PA hydrogel, indicating that high substrate stiffness jeopardized costamere expression of the hiPSC-CMs (Figures 4K,L).

## 4 Discussion

The multiple steps of myofibril growth are widely described as the models of myofibrillogenesis (Sanger et al., 2000). Newer results indicated that recruitment of Z-bodies during *de-novo* assembly of myofibrils was initialized at the integrin, vinculin, and talin-containing adhesion sites, which were termed as pre-costameres (Sparrow and Schöck, 2009). In mature myofibrils, the costameres, showing periodic rib-like patterns, were considered as the myofibril-sarcolemma connections registering with Z-lines and M-lines of the sarcomeres (ErvastiCostameres, 2003). From the observation of the pleat-like wrinkles forming on the flexible substrate during cardiomyocyte contraction, the costameres were proposed as the transmitters that mediated the force transmission from myofibrils to environmental ECM (Danowski et al., 1992). Despite the progress in elucidating details of the models of myofibrillogenesis and costamerogenesis, current understanding of how pre-costameres mature into costameres within the cardiomyocytes is still unclear. To address this gap in understanding, in previous work we documented costamere dynamics of the hiPSC-CMs during topography-induced cardiomyocyte reorganization (Shi et al., 2022). To better understand the process of costameres associated with different myofibril components (Z-lines and M-lines), in the present work we utilized hiPSC-CMs to profile the time-dependent costamerogenesis during cell attachment and spreading. By seeding hiPSC-CMs on PA hydrogel of different stiffnesses, we also studied how extracellular mechanical microenvironment affect myofibril and costamere structures.

### 4.1 Costamerogenesis and myofibrillogenesis occurred alternatively

Previous works indicated the irreplaceable role of costameres in the initial assembly of myofibrils (Chopra et al., 2018). In our study, though no distinguishable striated costameres were found at Hour 4-5, zyxin showed an early aggregation at the positions of Z-bodies in the pre-myofibrils and the interface between pre-myofibrils and nascent myofibrils. We believe that the *de-novo* assembly of pre-costameres participated in the recruitment of actinin to form the Z-bodies within pre-myofibrils and nascent myofibril at the early stage of myofibrillogenesis (Supplementary Figure S1, Figure 2B). It has been reported that in the later stage of costamerogenesis, the maturation of costameres relied on the integrity of mature myofibrils (Quach and Rando, 2006). The non-muscle myosin II diffusely existed in pre-myofibrils and nascent myofibrils but only aggregated near the bottom side of the cell membrane in cardiomyocytes with mature myofibrils, suggesting the non-muscle myosin may serve as the navigator in the cell-ECM interface to guide the maturation of costameres (Wang et al., 2018). In our research, both Z-costameres and M-costameres were assembled into an observable striated pattern at Hour 6, at which point the hiPSC-CMs show clear striated Z-lines and M-lines, indicating the maturation of myofibrils was prior to the maturation of costameres (Supplementary Figure S1, Figure 2B). We found that the Z-line distance and M-line distance showed no significant difference from Hour five to Hour 12, but the intensity of Z-costameres and M-costameres increased significantly during the same period (Figure 2 and Figure 3). This temporal alternation of costamere maturation and myofibril maturation suggests that the development of mature Z-lines and M-lines within the myofibrils is a prerequisite for costamere maturation.

### 4.2 Maturation of Z-costameres and M-costameres was asynchronous

The costameres registered with Z-lines were widely documented in previous cardiac studies, while the M-costameres have been rarely observed. In our previous study, we found that the ratio between costamere density and Z-line density within the hiPSC-CMs was higher than one but not equal to two, suggesting that the M-costameres and M-bands were not strictly paired. Moreover, we found that M-costameres were less detectable than the Z-costameres, suggesting that M-costameres were less expressed than the Z-costameres in hiPSC-CMs (Shi et al., 2022). In this study, we temporally quantified the intensity of Z-costameres and M-costameres after cell seeding to characterize the process of costamere maturation. We found that the intensity of Z-costameres and M-costameres increased in an alternating fashion, indicating the maturation of Z-costameres and M-costamere were asynchronous from Hour 12 to Hour 48 (Figures 3K,L). At Hour 48, the intensity of Z-costameres was significantly higher than M-costameres, suggesting that the Z-costameres were more predominant than the M-costameres in the hiPSC-CMs (Supplementary Figure S4).

### 4.3 Costameres of cardiomyocytes on substrates with different stiffness

It has been found that cardiomyocytes are able to adapt their contraction forces to environmental stiffening (Hersch et al., 2013). In our research, hiPSC-CMs on the 10 kPa substrates had higher force output than the ones on the 40 kPa substrates (Supplementary Figure S5), which was consistent with another study showing that hiPSC-CMs on stiffer substrates produced lower contractile force (Ribeiro et al., 2015). In addition, previous study reported that cardiomyocytes on a stiff substrate showed a decreased cell shortening (van Deel et al., 2017), which is consistent with our results showing a decrease in sarcomere shortening of hiPSC-CMs on 40 kPa PA hydrogel. We believe that the impaired sarcomere shortening may contribute to the impaired contractile function of hiPSC-CMs on 40 kPa PA hydrogel. It was also reported that cardiomyocytes on stiffer substrates developed disrupted myofibrils (Corbin et al., 2019). In this work, we found that the hiPSC-CMs on 40 kPa PA hydrogels showed shortened M-line length (Figure 4I), suggesting impairment of M-lines formation might lead to lower contractile force generation. In addition, we also found that both Z-line distance and M-line distance were increased in the hiPSC-CMs on the 40 kPa substrate (Figures 4C,D), which was consistent with previous work showing an increase of sarcomere length in a stiffer environment (Rodriguez et al., 2011). Showing a similar trend as the M-lines, M-costamere distance increased in the 40 kPa group (Figures 4D,J). This suggested that maturation of M-costameres required M-lines as the template. It has been reported that costameres function as the force transmitters during cardiomyocyte contractions (Danowski et al., 1992). It has been reported that costameres function as the force transmitters during cardiomyocyte contractions, while formation of mature striated costameres is intimately regulated by the contraction of cardiomyocytes (Danowski et al., 1992; Shakp et al., 1997). In our work, both Z-costameres and M-costameres showed lower intensity in contractile force-deficient hiPSC-CMs in the 40 kPa group (Figures 4K,L). We believe that the zyxin expression and costamere maturation does not directly respond to the substrate stiffness but might be mechanosensitive to the contractile force transmitted from sarcomeres to the extracellular matrices.

## 5 Conclusions and future work

In this study, we utilized hiPSC-CMs to study the assembly and maturation of costameres associated with the myofibrillogenesis processes. By temporally tracking the behaviors of costameres and myofibrils of the hiPSC-CMs, we found that costamerogenesis and myofibrillogenesis occurred in an alternating fashion. Early assembled pre-costameres participated in the recruitment of α-actinin to form pre-myofibrils and nascent myofibrils, leading to myofibril maturation. Next, using Z-lines and M-lines as templates in the myofibrils, pre-costameres grew into mature costameres with a striated pattern. During this process, the maturation of M-costameres and Z-costameres was asynchronous: Z-costameres showed a pattern of early formation but late maturation, while M-costameres showed a pattern of late formation but early maturation. By seeding hiPSC-CMs on PA hydrogels with different stiffness, we found that costamere maturation was regulated by the contractile function of cardiomyocytes. In future, the confocal microscopy with 3D Z-scanning of individual hiPSC-CMs with other components of costameres (e.g., integrin, vinculin, dystrophin) in a time-dependent manner will further enhance our understanding of how costameres incorporating with myofibrils would affect cardiomyocyte mechanobiology.

## Data Availability

The raw data supporting the conclusions of this article will be made available by the authors, without undue reservation.
