# Supplementary material for: Cooperation between myofibril growth and costamere maturation in human cardiomyocytes
Source: Front Bioeng Biotechnol. 2022 Nov 1;10:1049523. doi: 10.3389/fbioe.2022.1049523 (PMC9663467; doi:10.3389/fbioe.2022.1049523)
Supplement: Supplementary file 3 [file DataSheet1.pdf]

SUPPLEMENTARY INFORMATION

Cooperation between myofibril growth and costamere maturation in human cardiomyocytes

Huaiyu Shi[1,2], Chenyan Wang[1,2], Bruce Z. Gao[3], James H. Henderson[1,2], Zhen Ma[1,2*]

[1]Department of Biomedical & Chemical Engineering, Syracuse University, Syracuse, NY, USA
[2]BioInspired Institute for Materials and Living Systems, Syracuse University, Syracuse, NY, USA
[3]Department of Bioengineering, Clemson University, Clemson, SC, USA

*Corresponding author: Zhen Ma (zma112@syr.edu)

Table S1. Antibodies used in this study.

Antibodies	Vendors	Cat #	Dilution
Myomesin	DSHB	mMac myomesin B4	1:13
Zyxin	Sigma-Aldrich	HPA004835	1:200
647 goat anti mouse	Thermofisher	A32728	1:200
546 goat anti rabbit	Thermofisher	A11035	1:200

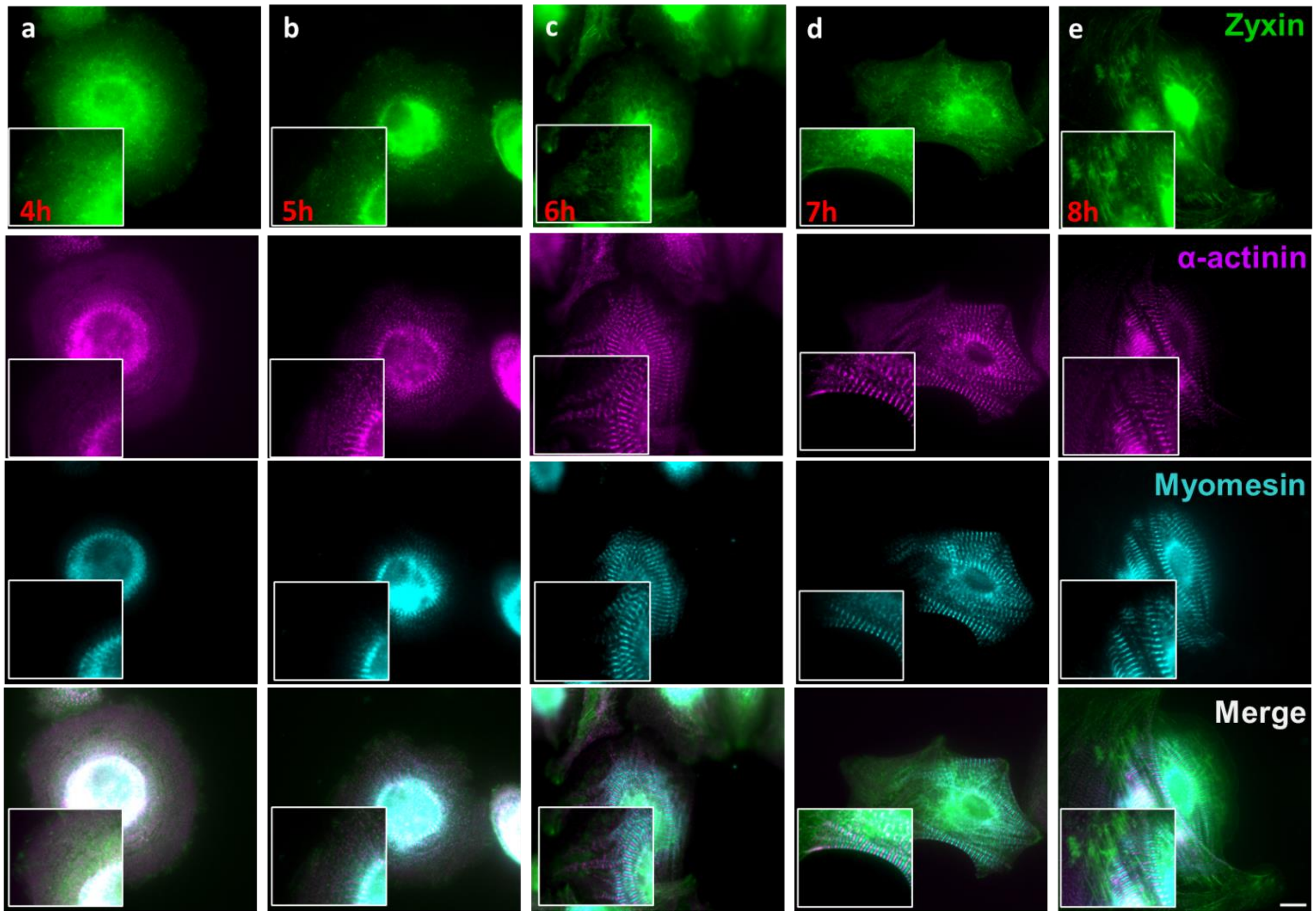

Figure S1. Zoom-in fluorescent images of hiPSC-CMs during Hour 4-8 after cell seeding. Fluorescent images of zyxin, myomesin and α-actinin co-staining at (a) Hour 4, (b) Hour 5, (c) Hour 6, (d) Hour 7 and (e) Hour 8. Scale bar: 10 μm

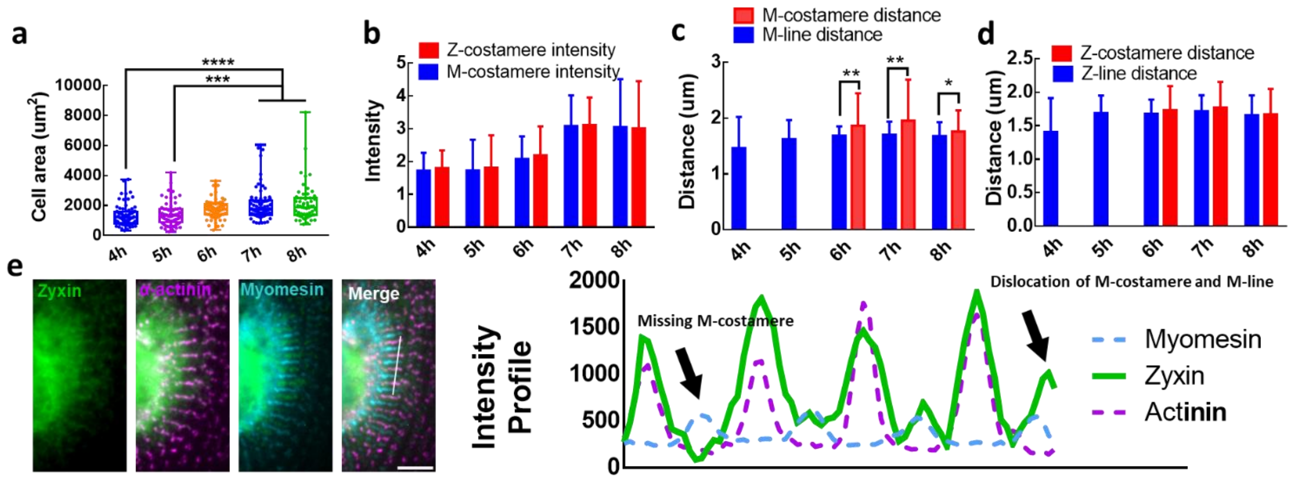

Figure S2. Assembly of costameres and myofibrils during cell attachment. (a) Cell area of hiPSC-CMs from Hour 4 to Hour 8 after cell seeding. (b) Comparison between Z-costamere intensity and M-costamere intensity. (c) The comparison between M-costamere distance and M-line distance. (d) Comparison between Z-costamere distance and Z-line distance. (e) Fluorescent images and intensity profiles indicated a missing M-costamere and the dislocation of M-lines and M-costameres. Scale bar: 5 µm. **p < 0.05, **p < 0.01, *** p < 0.001 and ****p < 0.0001*.

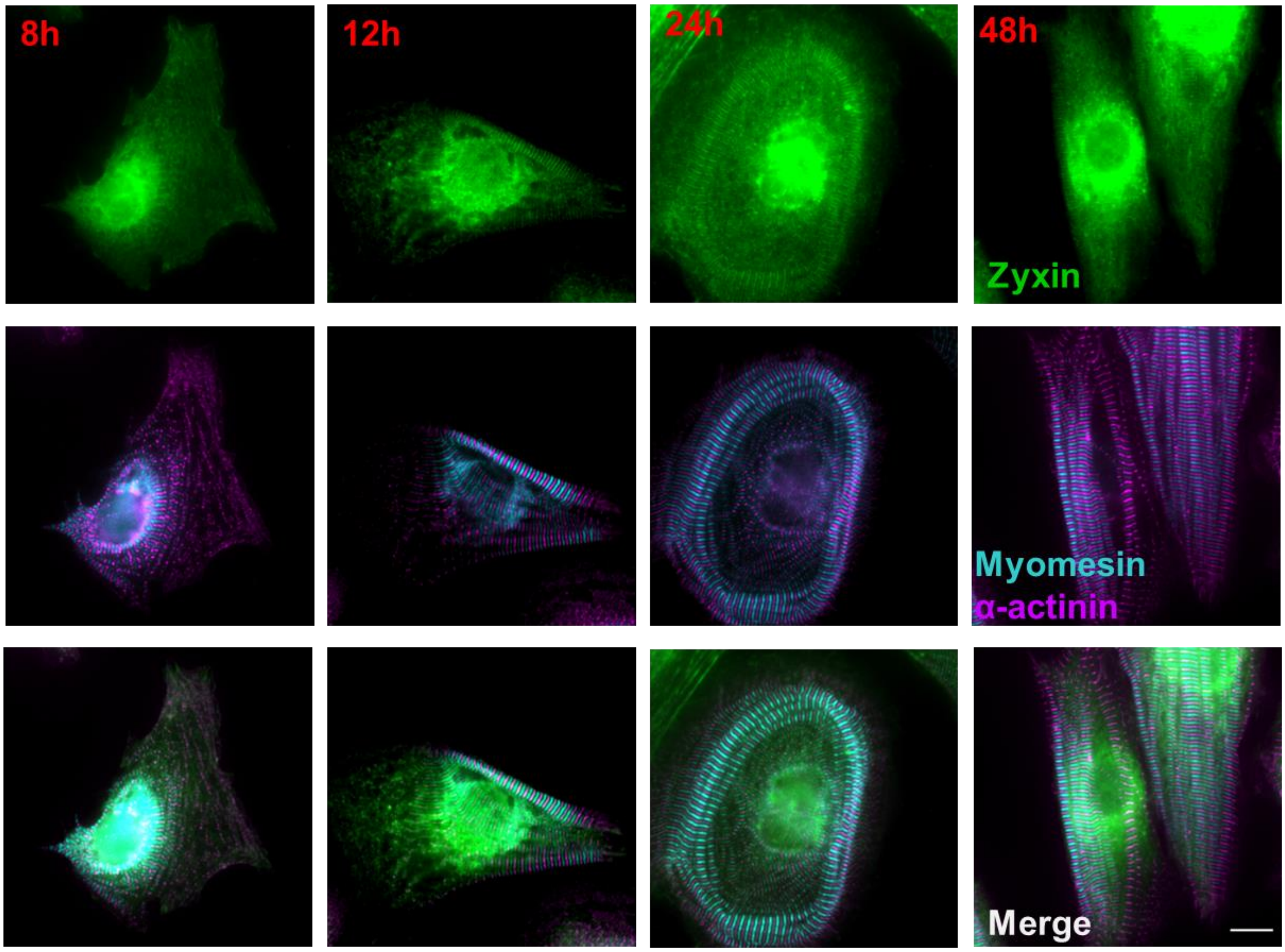

Figure S3. **Fluorescent images of hiPSC-CMs during cell spreading.** Fluorescent images of zyxin, myomesin and α-actinin co-staining at (a) Hour 8, (b) Hour 12, (c) Hour 24, and (d) Hour 48. Scale bar: 10 μm.

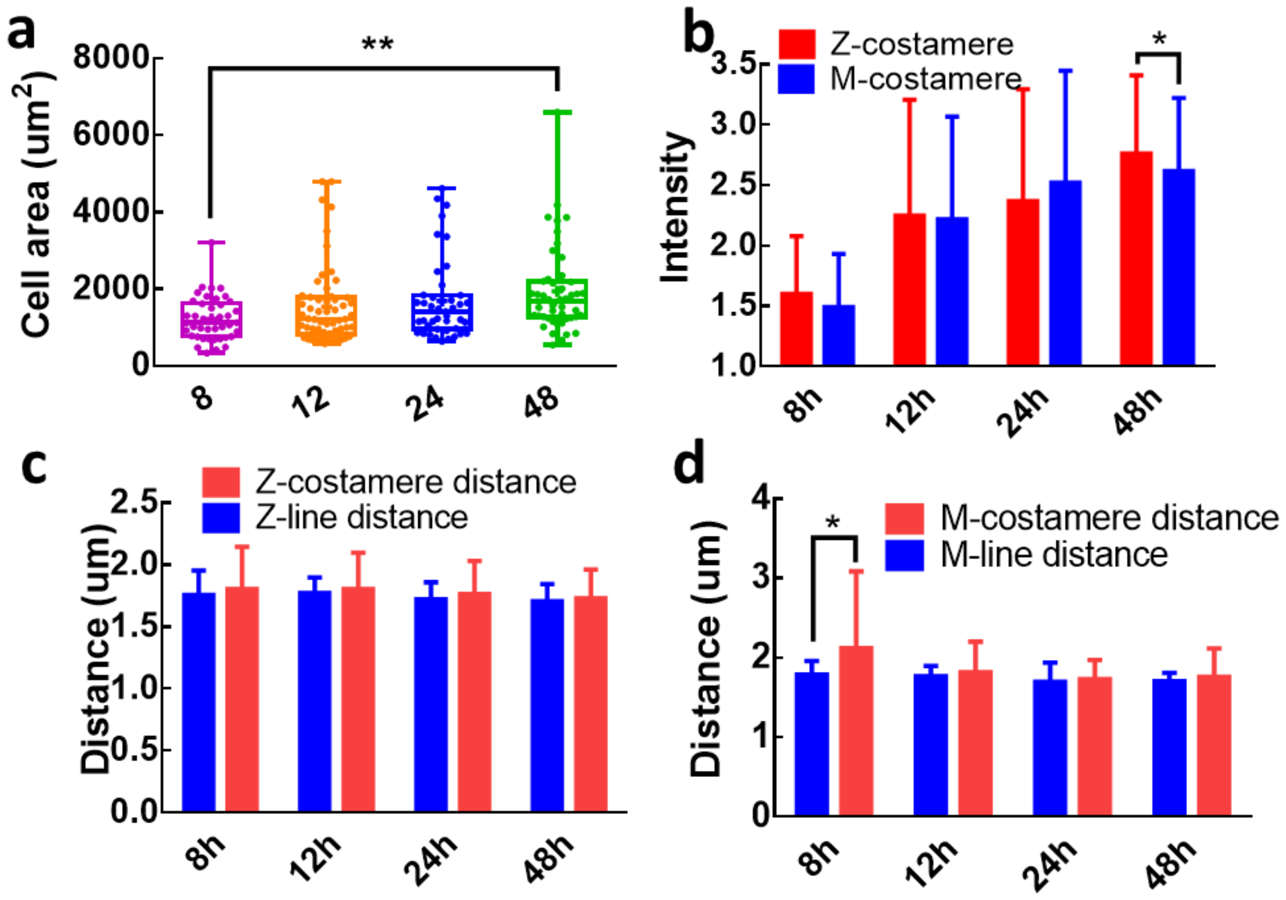

Figure S4. Maturation of costameres and myofibrils during cell spreading. (a) Cell area of hiPSC-CMs during cell spreading. (b) Comparison between Z-costamere intensity and M-costamere intensity. (c) Comparison between Z-costamere distance and Z-line distance. (d) Comparison between M-costamere distance and M-line distance. **$p < 0.05$, **$p < 0.01$, *** $p < 0.001$ and ****$p < 0.0001$.*

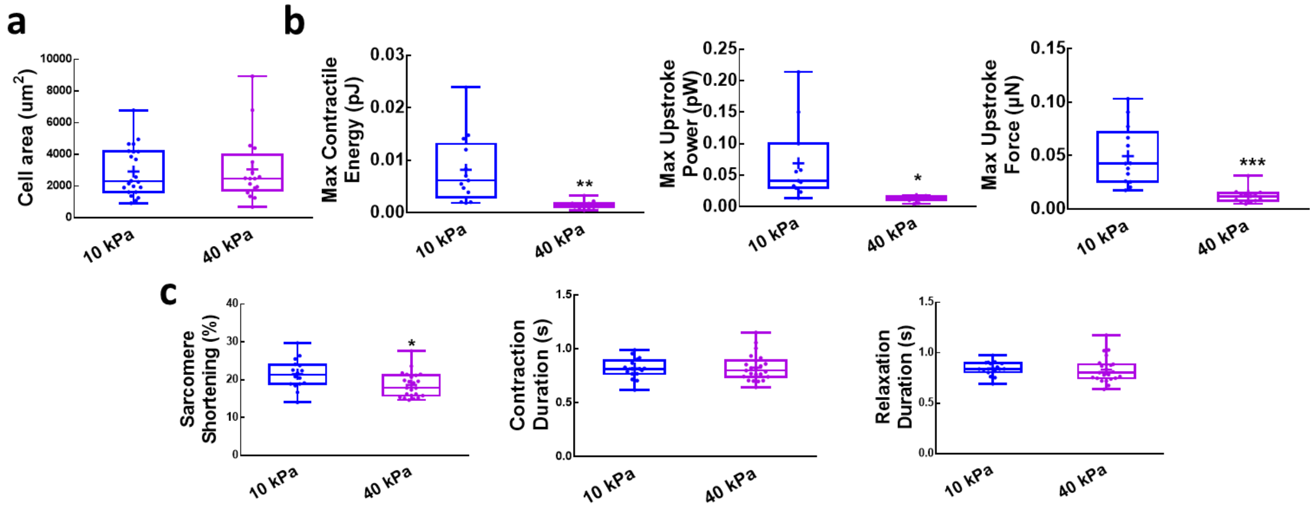

Figure S5. Costameres of hiPSC-CMs on different substrates. (a) Cell area of hiPSC-CMs on the PA hydrogels with 10 kPa and 40 kPa modulus. (b) The maximum contractile energy, maximum upstroke power and maximum upstroke force of hiPSC-CMs on the PA hydrogels with 10 kPa and 40 kPa modulus. (c) The sarcomere shortening, sarcomere contraction duration, sarcomere relaxation duration of hiPSC-CMs on the PA hydrogels with 10 kPa and 40 kPa modulus. *$p < 0.05$, **$p < 0.01$, *** $p < 0.001$ and ****$p < 0.0001$.

Movie S1. Video of moving dispersed fluorescent beads due to the contraction of a hiPSC-CM.

Movie S2. Heatmap video of contractile force generated by a hiPSC-CM.

Movie S3. Heatmap video of contractile energy generated by a hiPSC-CM.

Movie S4. Video of moving sarcomeres of a beating hiPSC-CM.
